# Perioperative transfusion threshold and ambulation after hip revision surgery – a randomized trial

**DOI:** 10.1186/1471-2253-14-89

**Published:** 2014-10-10

**Authors:** Kamilla Nielsen, Pär I Johansson, Benny Dahl, Michael Wagner, Britt Frausing, Jens Børglum, Kenneth Jensen, Jens Stürup, Jesper Hvolris, Lars S Rasmussen

**Affiliations:** Department of Anaesthesia, Centre of Head and Orthopaedics, Copenhagen University Hospital, Rigshospitalet, Kragujevac, Denmark; Section for Transfusion Medicine, Capital Region Blood Bank, Copenhagen University Hospital, Rigshospitalet, Kragujevac, Denmark; Department of Orthopaedic Surgery, Centre of Head and Orthopaedics, Copenhagen University Hospital, Rigshospitalet, Kragujevac, Denmark; Department of Occupational Therapy and Physiotherapy, Centre of Head and Orthopaedics, Copenhagen University Hospital, Rigshospitalet, Kragujevac, Denmark; Department of Anaesthesia and Intensive Care Medicine, Copenhagen University Hospital, Bispebjerg, Denmark; Department of Orthopedic Surgery, Copenhagen University Hospital, Bispebjerg, Denmark

**Keywords:** Transfusion threshold, Haemoglobin concentration, Red blood cells, Ambulation, Hip surgery

## Abstract

**Background:**

Transfusion with red blood cells (RBC) may be needed during hip revision surgery but the appropriate haemoglobin concentration (Hb) threshold for transfusion has not been well established. We hypothesized that a higher transfusion threshold would improve ambulation after hip revision surgery.

**Methods:**

The trial was registered at Clinicaltrials.gov ( NCT00906295). Sixty-six patients aged 18 years or older undergoing hip revision surgery were randomized to receive RBC at a Hb threshold of either 7.3 g/dL (restrictive group) or 8.9 g/dL (liberal group). Postoperative ambulation was assessed using Timed Up and Go-test (TUG) and ability to walk was also assessed daily by a physiotherapist blinded to the allocation.

**Results:**

Fifty-three patients were able to perform the TUG and included in the analysis. The TUG could be completed in a median of 36 sec vs. 30 sec in the restrictive group and the liberal group, respectively (P = 0.02). The mean difference in TUG was 14.5 sec (95% CI 2.8-26.2 sec). No difference was found in the day patients could perform TUG or walk 10 meters. The Hb at the day of testing was 10.2 g/dL in the restrictive group and 9.9 g/dL in the liberal group. Only 26 patients received RBC.

**Conclusions:**

A Hb transfusion threshold of 8.9 g/dL was associated with a statistically significantly faster TUG after hip revision surgery compared to a threshold of 7.3 g/dL but the clinical importance is questionable and the groups did not differ in Hb at the time of testing.

## Background

Orthopaedic surgery may be associated with significant blood loss and transfusion with allogeneic red blood cells (RBC) may be needed. Postoperative anaemia is suspected to impair mobilization but the appropriate haemoglobin concentration (Hb) threshold for transfusion of RBC has not been well established. A relatively high transfusion threshold may enhance postoperative ambulation but potential side effects, immunologic as well as non-immunologic, are reasons not to administer allogeneic RBC liberally [1, 2]. Increased morbidity and mortality has been found in a recent analysis including more than 10,000 surgical patients who received RBC transfusion [3]. In contrast, no significant association has been found between RBC transfusion and 30- or 90-day mortality in a large study [4]. A Cochrane review on RBC transfusion thresholds identified 19 randomized trials including a total of 6,264 patients. It suggested that hospital mortality was 23% lower in patients in the restrictive compared to the liberal transfusion strategy (RR0.77, 95% CI 0.62 to 0.95) and concluded that a restrictive transfusion threshold is safe in patients without serious cardiac disease [5].

The American Society of Anesthesiologists recommends transfusion of RBC to patients without cardiac disease at a haemoglobin concentration of 6 g/dL [6]. Often patients are not transfused according to these guidelines [4, 7–9]. This could be due to concerns about early recovery since anaemia in the immediately postoperative period could impede mobilization and prolong hospital stay [10–12]. In a study of young healthy volunteers increased level of fatigue was reported during progressive isovolaemic haemodilution to a nadir Hb of 5 g/dL [13], but such data are not easily transferred to the often older and frailer patients undergoing major joint surgery. No difference in mortality or the ability to walk at the 60-day follow-up was found in a recent large randomized trial in an elderly population with cardiovascular risk factors or cardiac disease [14]. The aim of this study was to compare the influence of two transfusion strategies on ambulation in patients undergoing major joint surgery.

We hypothesized that a higher transfusion threshold would improve ambulation after hip revision surgery.

## Methods

The Committees on Biomedical Research Ethics of the Capital Region of Denmark and the Danish Data Protecting Agency approved the trial that was registered at Clinicaltrials.gov ( NCT00906295). Written informed consent was obtained before surgery from all patients. Patients were eligible if they were at least 18 years of age and scheduled for elective hip revision surgery. Exclusion criteria were disseminated cancer or cardiac disease with functional impairment (NYHA class II or above). Recruitment took place in the ward the day before surgery or at the preanaesthetic assessment prior to surgery. The randomization was done preoperatively after informed consent was obtained. A dedicated computer program (Idefix) was used after entering patients’ baseline data. Only one investigator had access to the program. Investigators at the other hospital had to call this investigator to randomize. The allocation was written on a form which was kept in the investigator’s office and the allocation could only be accessed by the investigator in charge of administrating red blood cells. The patients were randomized to a restrictive strategy receiving transfusion of RBC at a Hb of 7.3 g/dL (4.5 mmol/L) or a liberal strategy receiving transfusion of RBC at a Hb of 8.9 g/dL (5.5 mmol/L). The target level of haemoglobin in the restrictive group was 7.3 to 8.9 g/dL and above 8.9 g/dL in the liberal group. The Danish National Board of Health recommends transfusion of allogeneic RBC at a Hb of 7.3 g/dL, in patients free of cardiac disease [15]. The lowest transfusion threshold was therefore set at 7.3 g/dL. The transfusion threshold of the liberal group was set at 8.9 g/dL since a higher level was likewise considered unethical.

During surgery, monitoring included pulse oximetry, ECG, blood pressure (BP) measurement, and urinary output. Patients were warmed by forced-air patient warming system and core temperature was monitored. General anaesthesia was administered using propofol or sevoflurane with remifentanil or fentanyl. Spinal anaesthesia was used if requested by the patient and accepted by the attending anaesthesiologist. Tinzaparin 3,500 IE was given subcutaneously daily as thromboprophylaxis from the evening before surgery and until discharge from hospital. Two grams of tranexamic acid were administered intravenously after surgical incision to minimize blood loss. No blood salvage techniques were used and no optimization of Hb before surgery was done. Arterial blood gases were drawn to guide decisions about transfusion and always at the end of surgery. We recorded Hb, blood loss, urinary output, fluids administered, and transfusions of blood products during surgery and in the post anaesthesia care unit (PACU). If the patients had returned to the ward before evening rounds an additional Hb was measured on the day of surgery. Hb, pulse, BP, fluids and RBC administered were noted daily from the day following surgery and until testing of ambulation. Pain was evaluated daily with a Visual Analogue Scale (VAS) from 0–100 both at rest and while actively bending the knee and hip to a maximum of 90 degrees. These measurements were performed during weekdays.

### Fluid therapy

Infusion of Ringers lactate or isotonic saline was started before induction of anaesthesia and maintained during surgery to replace urinary loss. Blood loss greater than 500 mL was replaced with the same volume of HES *130*/0.4 (Voluven®, Fresenius Kabi Canada 45 Vogell Road, Suite 2010 Richmond Hill, Ontario L4B 3P6). Additional fluids were administered if mean arterial pressure (MAP) decreased during or after surgery. If MAP decreased more than 25% during surgery ephedrine, phenylephrine or dopamine was administered. Fluid loss in the ward was replaced orally. Additional Ringers lactate, isotonic saline or HES 130/0.4 could be given if hypovolaemia or severe dehydration was suspected.

### Pain management

All patients had one gram of paracetamol administered four times a day. The standard regimen included patient controlled analgesia (PCA) using a bolus of 4 mg morphine as needed and a continuous intravenous infusion of morphine 1 mg per hour and continued in the ward until standardized oral pain treatment with paracetamol and morphine was initiated. Patients who had an epidural catheter were given a continuous epidural infusion of a mixture consisting of ropivacain1.25 mg with morphine 50 μg per mL, 5 mL per hour. The epidural catheter was kept for a maximum of three days postoperatively. Local Infiltration Analgesia (LIA) could be given as infiltration of a mixture of 100 mL ropivacain 2 mg/mL, ketorolac 15 mg, and epinephrine 0.25 mg given at the end of surgery with additional boluses given eight and 16 hours postoperatively via a catheter placed within the joint capsule.

In the PACU pain treatment could be supplemented with intravenous morphine, ketobemidone 5 mg or fentanyl 50 μg as needed.

### Physiotherapy and rehabilitation

The rehabilitation program began the day after surgery. The patients were consulted daily on weekdays for half an hour by a physiotherapist. Patients were instructed in range of motion as well as weight bearing and exercises to improve vein flow. Isometric as well as active muscle training were started and transferring and rising from bed trained as well. Walking was initiated the first day after surgery using a high-walker. Walking ability and the need for appropriate walking-aids were evaluated and from the second day this rehabilitation became progressively more active.

### Intervention

Transfusion of allogeneic RBC was administered according to allocation but the attending orthopaedic surgeon could decide to give additional transfusion of RBC as a safety precaution. In that situation, the investigator had to be notified and a reason for transfusing was documented. Fresh frozen plasma was given along the seventh unit of RBC or if laboratory tests indicated it.

### Endpoints

The primary outcome measure was the Timed Up and Go-test [16]. TUG was used to assess the time it takes a patient to stand up, walk three meters, turn around, walk back and sit down again. On the command "ready-walk" the patient rises from a chair and walks to a line on the floor. At least one foot has to touch the line. The patient was encouraged to do the test as fast as possible. No support from another person was allowed during testing but verbal guidance could be given. The procedure was timed by the physiotherapist and no practice trial was done. TUG was performed on the day patients were considered able to walk at least six meters and rise from a chair.

Secondary outcome measures were the day the patients could perform TUG, the day a 10 meter walking distance was reached, and length of hospital stay. It was recorded if patients were only allowed partial weight bearing on the operated leg, had an epidural or used walking aids during TUG.

The patient’s medical files were scrutinized to assess postoperative complications within 30 days after surgery.

The allocation and Hb during the testing period were concealed from the patients but the investigator, the staff in the operating room, and at the staff at the ward could not be blinded. The physiotherapist testing the patient was blinded. The operating room staff, the PACU nurse and the ward nurses were informed not to reveal the allocation or Hb. Stickers in the patient’s medical file informed the staff that the patient participated in the trial and therefore should not be informed about Hb. Patients were seen every weekday by another physiotherapist than the one responsible for ambulation. Two separate case report forms were used, one set for the investigator and another for the physiotherapist testing the patient. The case report forms were kept separately from the patients file. The analyses of the primary and secondary endpoints were done blindly by concealing the allocation during the statistical analysis.

### Statistics

Patient characteristics and perioperative variables were reported using median and interquartile range. Mann-Whitney’s test was used to compare TUG and Chi square test used to compare day for TUG and day for 10 meter walking distance. A two-sided probability value of less than 0.05 was considered significant.

A difference in TUG of 15 seconds was considered clinically relevant. A former study found a SD for TUG of 17.6 seconds in elderly patients undergoing total hip replacement [17]. A sample of 52 patients was considered necessary to detect a difference of 15 seconds with a power of 85% at the 5% significance level. Statistical analyses were performed with SAS 9.1 for Windows.

## Results

**Figure 1:**
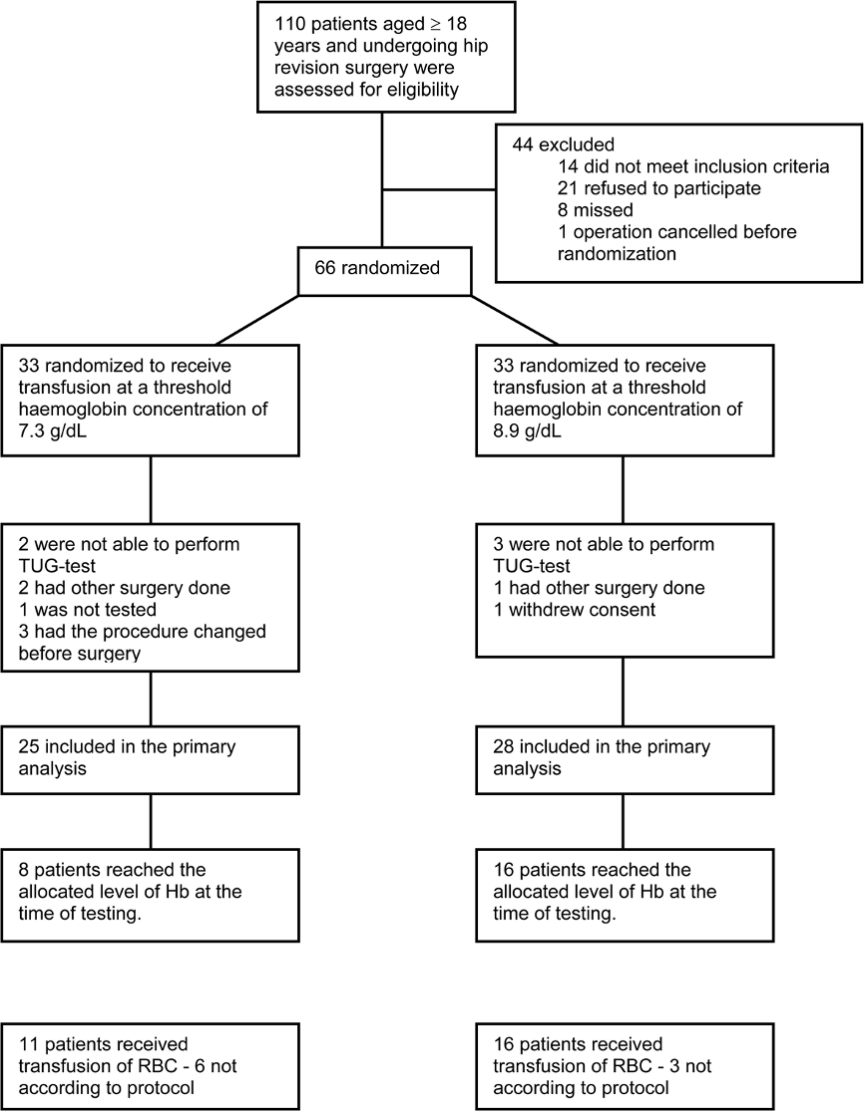
**Patient flow.** Patient enrolment, randomization and treatment flow.

Sixty-seven patients were enrolled between June 22, 2009 and May 13, 2011 (Figure 1). One patient had surgery cancelled before randomization. A total of 66 patients were randomized. Fifty-five patients were included at Rigshospitalet and twelve patients at Bispebjerg hospital. Demographic characteristics were similar between the groups (Table 1).

**Table 1:** **Characteristics, median with (5-95**% **range), or number with proportion**

***Characteristics***	Restrictive group (n = 33)	Liberal group (n = 33)
**Age, years**	68 (43–86)	72 (53–89)
**Men/women, no. (% men)**	16/17 (48)	20/13 (61)
**Body mass index, kg/m** ^**2**^	27 (20–39)	26 (20–34)
**American Society of Anesthesiologists physical status score, no. (%)**		
1	5 (15)	6 (18)
2	26 (79)	22 (67)
3	2 (6)	5 (15)
**Comorbidities, no. (%)**		
Current smoker	4 (12)	5 (15)
Diabetes	6 (18)	4 (12)
Hypertension	18 (55)	19 (57)
Other cardiovascular disease	5 (15)	7 (21)
Lung disease	2 (6)	3 (9)
Neurologic disease	1 (3)	2 (6)
Kidney disease	1 (3)	0
Other disease	8 (24)	8 (24)
Preoperative haemoglobin concentration, g/dL	13.4 (10.2-15.0)	13.8 (10.5-16.3)

Patient characteristics of patients undergoing hip revision surgery and randomized to a transfusion threshold of red blood cells of a haemoglobin concentration of 7.3 or 8.9 g/dL, restrictive group and liberal group, respectively.

**Figure 2:**
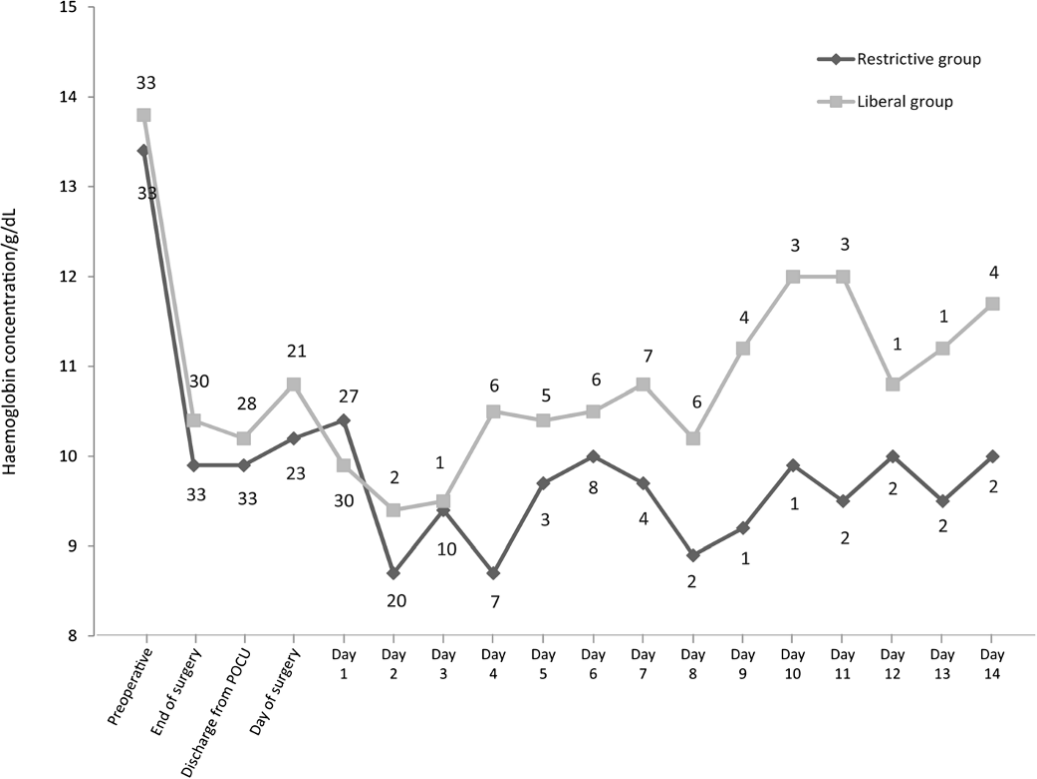
**Haemoglobin concentration.** Median haemoglobin concentration in patients undergoing hip revision surgery and randomized to a transfusion threshold of red blood cells of a haemoglobin concentration of 7.3 or 8.9 g/dL, restrictive group and liberal group, respectively. Plotted from before surgery until TUG was performed or patients were discharged from hospital. The number of patients is shown with digits above the graphs.

Perioperative blood loss, fluid therapy, and Hb values are displayed in Table 2 and Figure 2. The median perioperative blood loss was 575 mL and 900 mL in the restrictive group and the liberal group, respectively (P = 0.10). A total of eleven patients in the restrictive group and sixteen in the liberal group received transfusion of RBC. Six patients in the restrictive group received eleven units of RBC not according to protocol. In the liberal group three patients received five units of RBC not according to protocol (P = 0.28). One patient in each group had gastric bleeding necessitating RBC transfusion. Other indications for giving transfusions not according to allocation included fatigue, dizziness, pallor, nausea, dyspnoea, and atrial fibrillation. The distribution of postoperative complications was similar as to the number of patients who experienced complications. Thirty-two complications were registered in the restrictive group and 54 in the liberal group (0.01) (Table 3).

**Table 2:** **Perioperative variables, median with (5-95% range), or number with proportion**

	Restrictive group (n = 33)	Liberal group (n = 33)	P-value
***Surgery***			
**Type of surgery, no. (%)**			
Total revision	13 (43)	15 (45.5)	0.05
Acetabular component revision	9 (30)	15 (45.5)	
Femoral component revision	6 (20)	2 (6)	
Other	2 (7)	1 (3)	
**Type of anesthesia, no. (%)**			
General anaesthesia	26 (87)	31 (94)	0.41
Spinal anaesthesia	4 (13)	2 (6)	
**Pain management, no. (%)**			
Femoral nerve block	2 (7)	1 (3)	0.60
Local infiltration analgesia with catheter	2 (7)	1 (3)	0.60
Local infiltration analgesia without catheter	8 (27)	10 (30)	0.79
Epidural as postoperative pain management	11 (33)	17 (52)	0.31
**Blood loss during surgery, mL**	575 (150–2300)	900 (200–2900)	0.10
**Urinary output during surgery, mL**	340 (76–975)	300 (65–1000)	0.84
**Fluids given during surgery, mL** ^**1**^			
Red Blood Cells	0 (0–490)	0 (0–980)	0.05
Crystalloids	1500 (500–3500)	1500 (300–3100)	0.64
Colloids	500 (0–1500)	750 (0–2000)	0.11
**Last Hb measured during surgery, g/dL**	9.9 (6.5-12.8)	10.4 (8.3-12.9)	0.31
***Post Anaesthesia Care Unit (PACU)*** ^***1***^			
**Blood loss in drain if used, mL**	150 (0–700)	150 (0–500)	0.73
**Urinary output during stay at PACU, mL**	121 (49–190)	260 (45–1000)	0.70
**Transfusions of red blood cells in PACU, mL**	0 (0–245)	0 (0–245)	0.43
**Crystalloids given in PACU, mL**	700 (200–1200)	1000 (200–2400)	0.03
**Colloids given in PACU, mL**	0 (0–500)	0 (0–500)	0.93
**Haemoglobin concentration at discharge from PACU, g/dL**	9.9 (7.9-12.0)	10.2 (8.9-14.4)	0.16
***Postoperative period***			
**Crystalloids given, mL**	0 (0–4000)	500 (0–4000)	0.66
**Colloids given, mL**	0 (0–500)	0 (0–1500)	0.25
**Total perioperative transfusions of red blood cells, mL**	0 (0–1470)	245 (0–1225)	0.22
**Total number of patients transfused, no. (%)**	11 (37)	16 (48)	0.32

Mean where indicated.

Type of surgery, anesthesia, and perioperative fluid and pain management of patients undergoing hip revision surgery and randomized to receive transfusion of red blood cells at a haemoglobin concentration of 7.3 or 8.9 g/dL, restrictive group and liberal group, respectively.

^1^One patient in the liberal group was transfused 1,100 mL of fresh frozen plasma (FFP) during sugery and one patient in the restrictive group was transfused 275 mL of FFP in the Post Anasthesia Care Unit. No further transfusions of FFP was given nor did any patients receive transfusion of platelets during the trial.

**Table 3:** **Postoperative complications**

	Restrictive group (n = 30)	Liberal group (n = 33)	P value
Gastrointestinal complications	12	13	
Pneumonia	0	4	
Other pulmonary	2	3	
Skin reaction	3	0	
Fever	5	3	
Dizziness/discomfort	4	8	
Hypotension	1	4	
Fatigue	1	1	
Hyperkalemia	1	1	
Deep vein thrombosis	0	1	
Surgical site infection	0	1	
Fall at the ward or at home	3	2	
Others	3	16	
**Total number of patients experiencing postoperative complications**	19	22	0.61
Patients experiencing one postoperative complication	9	6	
Patients experiencing two postoperative complications	5	6	
Patients experiencing three postoperative complications	4	4	
Patients experiencing four postoperative complications	1	3	
Patients experiencing five postoperative complications	0	3	
**Total number of postoperative complications**	34	57	0.01

Postoperative complications 30 days after surgery in patients undergoing hip revision surgery and randomized to receive transfusion of red blood cells at a haemoglobin concentration of 7.3 or 8.9 g/dL, restrictive group and liberal group, respectively.

Fifty-three patients were able to perform TUG. The median time for TUG was 36 (5-95% range: 16–57) and 30 seconds (5-95% range: 23–87) in the restrictive and liberal group, respectively (P = 0.02). TUG was performed after a median of 2.0 days (restrictive group) and 2.5 days (liberal group) after surgery (P = 0.81). Both groups were able to walk 10 meters at a median of two days after surgery (P = 0.81) (Table 4). Median length of hospital stay was eight days in both groups.

**Table 4:** **Functional outcomes and pain scores, median with (5-95**% **range), or number with proportion**

	Restrictive group	Liberal group	P value
**Time for Timed Up and Go test, seconds**	36 (16–57) (n = 25)	30 (23–87) (n = 28)	0.02
**Postoperative day patients could perform TUG**			
1	5	5	0.87
2	8	9	
3	3	3	
4	2	5	
≥ 5	7	6	
**Postoperative day patients were able to walk 10 meters**			
1	11	12	0.93
2	11	10	
≥3	4	7	
**Patients TUG tested with epidural catheter, no. (%)**	2 (8) (n = 25)	4 (14) (n = 28)	0.67
**Pain score on the day of testing**			
VAS-score at rest^*^	0.5 (0–29) (n = 24)	0 (0–48) (n = 25)	0.86
VAS-score at activity^†^	20.5 (0–67) (n = 24)	22 (0–75) (n = 25)	0.79
**Hb at the day of test, g/dL**	10.2 (8.1-11.8) (n = 23)	9.9 (8.3-12.5) (n = 28)	0.87
**Length of hospital stay after surgery, days**	8 (4–44) (n = 33)	8 (4–38) (n = 33)	0.71
**Allowed only partial weight bearing, no. (%)**	12 (48)	14 (50)	1.00
**Type of walking aid, no. (%)**			
Crutches	11 (44)	11 (39)	0.11
Zimmer frame	1 (4)	3 (11)	
Walking frame	6 (24)	3 (11)	
Highwalker	7 (28)	11 (39)	

Postoperative functional outcome measures and pain scores for patients undergoing hip revision surgery and randomized to a transfusion threshold of red blood cells of a haemoglobin concentration of 7.3 or 8.9 g/dL, restrictive group and liberal group, respectively.

^*^Visual Analogue Scale score assessed from 0 to 100 during rest.

^†^Visual Analogue Scale score assessed from 0 to 100 while actively flexing the knee to a maximum.

Twenty-four of the 66 randomized patients reached the allocated level of Hb at the time of testing; eight patients in the restrictive group and 16 in the liberal group. No difference in the time to perform TUG was found in this subgroup.

All patients were alive at the 30-day follow-up.

## Discussion

In patients undergoing hip revision surgery, a Hb transfusion threshold of 8.9 g/dL was associated with a statistically significant faster postoperative TUG than a threshold of 7.3 g/dL. However, the difference between the median values was only six seconds, and there was no difference regarding how soon after surgery the patients could perform TUG or walk 10 meters. Not surprising, more patients received RBC transfusion in the liberal group and more patients in the restrictive group were transfused not according to protocol.

Several limitations in this study have to be discussed. Firstly, Hb decreased by approximately 25% after surgery in both groups and the transfusion threshold was never reached in a considerable number of patients, which minimized the potential difference between the groups. Secondly, the median Hb was 10.2 g/dL and 9.9 g/dL in the in the restrictive and liberal group, respectively, on the day of TUG testing. The small difference in TUG is therefore not likely related to a difference in Hb and the clinical importance is highly questionable.

The detected difference in TUG was much smaller than the 15 seconds we considered clinically relevant. The sample size calculation was based on data that seemed to be of limited relevance for our specific patient group but that was not known when the trial was planned.

The minor difference in Hb between the two groups was related to the fact that intraoperative blood loss was quite limited. The perioperative blood loss tended to be greater in the liberal group although not statistically significant. Nonetheless, the greater blood loss in the liberal group mades it difficult to achieve a difference in Hb between the groups. Likewise, more fluid was administered in the liberal group. Thirdly, we included patients scheduled for revision surgery of the hip, regardless of the indication for surgery, making our cohort somewhat heterogeneous in terms of surgical procedure and to what extent patients could be mobilized (Table 2). Finally, the type of anaesthesia was not strictly standardized and several different regimens of postoperative pain relief were employed. In particular, lumbar epidural analgesia may cause motor paralysis of the lower extremities which may influence ambulation. Pain was not evaluated in detail at the time of testing and this may affect the TUG-score but pain intensity was generally low and not different between groups. The different types of walking aids used could also affect the time it takes to do perform the TUG. Other limitations include that the TUG was performed without a practice trial as often recommended and that the TUG-testing took place on weekdays only. The latter was due to the limited availability of physiotherapy during weekends, which potentially could obscure the effect of the intervention. Given these limitations, it is difficult to say whether a liberal transfusion strategy is indeed advantageous for mobilization after major hip surgery but the findings in our trial do not firmly support a liberal transfusion strategy.

The Danish National Board of Health recommends transfusion of allogeneic RBC at a Hb of 7.3 g/dL, in patients free of cardiac disease [15]. The lowest transfusion threshold was therefore set at 7.3 g/dL. Compared to guidelines issued by The American Society of Anaesthesiologists it can be argued that the lower limit was too high. The transfusion threshold in Denmark is, however, at that level and a lower limit than the one issued by the national authorities was considered unethical.

The total number of complications were larger in the liberal group and more patients experienced complications in this group, although not significantly more. The trial was not designed to investigate complications of RBC transfusion. Still, some pulmonary complications may be related to TRALI, a known complication to transfusion. This would support a restrictive strategy of transfusion.

Our trial did not address whether a higher transfusion threshold could have any long-term effect since patients were tested within the third day of surgery.

A higher level of haemoglobin could increase the amount of oxygen delivered to the tissues. Accordingly, symptoms of anaemia, such as impaired mobilization, should be alleviated. Still, the trials conducted so far have shown contradicting results [10, 11, 18–31], and the majority is of observational design [10, 11, 18–24] why further research is warranted. The results of our trial indicate that perioperative blood loss in hip revision surgery infrequently requires RBC transfusion, probably as a result of improved surgical techniques and the use of fibrinolytic medication. Previous trials have also experienced challenges similar to the ones encountered in ours. Several lessons can be learned from our study and should be taken into consideration by investigators who want to study transfusion thresholds and ambulation. Firstly, the type of surgery should be considered thoroughly. It should be an operation with the potential of early postoperative ambulation as well as a substantial loss of blood. Secondly, the risk of protocol violation must be minimized by a strict adherence to the protocol, especially in trials conducted over the postoperative period where numerous medical doctors will be involved patient care. Perioperative blood loss is impossible to predict and a large proportion of patients will only have a minor blood loss and no need for transfusion of RBC. Another major concern in transfusion medicine is the need for a "golden standard" in measuring ambulation and walking ability. Different studies uses different outcome measures making comparison between trials problematic.

Our study may be considered a feasibility study that illustrates the significant challenges in studying transfusion thresholds, especially related to compliance with the protocol and as such, it can hopefully provide the basis for new prospective studies in this field.

## Conclusions

In conclusion, a Hb transfusion threshold of 8.9 g/dL was associated with a statistically significantly faster TUG after hip revision surgery than a threshold of 7.3 g/dL but the clinical importance is questionable and the groups did not differ in Hb at the time of testing.

## Abbreviations

BP: Blood pressure
FFP: Fresh frozen plasma
Hb: Haemoglobin concentration
LIA: Local infiltration analgesia
MAP: Mean arterial pressure
PACU: Postoperative care unit
PCA: Patient controlled analgesia
RBC: Red blood cells
TUG: Timed up and go-test
VAS: Visual analogue scale.
